# Periodontal Disease and Grip Strength among Older Adults

**DOI:** 10.3390/geriatrics5030046

**Published:** 2020-08-26

**Authors:** Vinish Aravindakshan, Faisal F. Hakeem, Wael Sabbah

**Affiliations:** 1 Faculty of Dentistry, Oral, & Craniofacial Sciences, King’s College London, London SE5 9RW, UK; Vinish_aravindakshan@hsdm.harvard.edu (V.A.); Wael.Sabbah@kcl.ac.uk (W.S.); 2 Harvard School of Dental Medicine, Boston, MA 02115, USA; 3 Department of Preventive Dental Sciences, College of Dentistry, Taibah University Dental College & Hospital, Madinah 42353, Saudi Arabia

**Keywords:** frailty, musculoskeletal, older adults, oral health, periodontal disease

## Abstract

**Objective:** The aim of this research was to assess the association between periodontitis and grip strength among older American adults. Methods: Data from the National Health and Nutrition Examination Survey 2011/2012 and 2013/2014 were used. Oral health status and hand grip strength were clinically assessed. Three outcome variables were used: (1) handgrip strength <30 kg for men, <20 kg for women; (2) handgrip strength <26 kg for men, <16 kg for women; and (3) mean maximum grip strength. The main exposure was the case definition of periodontitis. Logistic and linear regression models were constructed for grip strength definitions and the mean grip strength, respectively, adjusting for covariates. Results: The study included 1953 participants. The mean age was 68.5 years, and 47.2% were males. The prevalence of low grip strength (<30 kg for men, <20 kg for women) was 7.4% in men and 13.6% in women. Periodontitis was significantly associated with grip strength (OR 1.53, 95% CI: 1.03, 2.27) in the unadjusted model. Periodontitis was also significantly associated with maximum grip strength (Coefficient 1.05, 95% CI −1.99, −0.09) in a model adjusted for age and gender. However, in all the fully adjusted models there was no statistically significant association between periodontitis and grip strength. Conclusion: Low grip strength appeared to be more common among persons with moderate/severe periodontitis. The observed association is probably attributed to older age and common risk factors for periodontitis and frailty.

## 1. Introduction

There is a steep increase in the ageing population worldwide with the number of adults aged 60 years or more estimated to become 2 billion by the year 2050 [1]. The number of older adults in the USA is estimated to reach more than 80 million by the year 2030 [2]. This rapidly growing older population has become a major concern for public health as it is linked to the progression of different geriatric conditions. Prominent among these is frailty, which is linked to several adverse health outcomes [3]. It is worth noting that among the global population of older adults above 85 years, about 25% to 50% are frail [4].

Frailty is “an age-related medical syndrome which results in the decline in many physiological systems including musculoskeletal function, decreased functional reserve capacity and vulnerability against minor stressors” [5]. The functional decline during the onset of frailty and the loss of muscle mass and strength can significantly decrease the “Quality of life” of older adults [6]. These implications of frailty are reflected in poorer health outcomes and an increase in healthcare expenditure [7]. As a result, it is of utmost importance to deepen our understanding of frailty and its risk factors.

Growing evidence indicates that oral health is associated with physical decline and frailty at old age [8,9,10]. Low hand grip strength is an important indicator of physical decline at old age and one of the criteria of the frailty phenotype [11]. Previous studies have found significant associations between oral health and handgrip strength among older adults [12,13,14,15]. However, these studies mainly focused on the number of remaining teeth and the use of dental prothesis and they did not assess the role of periodontal diseases. Periodontal disease can result in tooth loss and is associated with many general health conditions [16]. Studies have shown that with periodontal disease, there is an increase in inflammation markers, like IL-6 (Interleukin 6) and C-reactive protein (CRP) which were related to decreased physical capability and muscle strength in frail adults [17].

Even though previous studies have examined the association between oral health and grip strength, we are not aware of any study that has assessed the association between periodontal disease and grip strength in a nationally representative sample of older adults. The aim of this study is to examine the association between moderate/severe periodontitis and handgrip strength, and to assess whether this association, if existed, is independent from other risk factors.

## 2. Materials and Methods

### 2.1. Study Population

We used data from the National Health and Nutrition Examination Survey (NHANES) 2011–2012 and 2013–2014, a cross-sectional study of a nationally representative sample of the non-institutionalized USA population. These surveys were carried out using a complex and multi-stage probability sampling framework. Initially, primary sampling units (PSUs) were selected, which were later divided into segments. Eventually, certain households were chosen from these segments and a selection of study participants was carried out [18]. The analysis included a total of 2216 adults aged ≥60 years from the NHANES 2011–2014 surveys. Out of the participants who were eligible, a total of 1953 older adults finished the interviews and examinations. Those who had missing information on oral health status, grip strength, medical conditions and education were excluded from the study sample [19].

### 2.2. Measurements

NHANES 2011–2012 and 2013–2014 consisted of interviews carried out by trained interviewers at households or Mobile Examination Centers (MEC). Clinical examinations, which comprised of assessments of grip strength evaluation, Body Mass Index (BMI) and oral health, were held at MECs. During oral health examination, data were documented by independent recorders. Personnel who conducted the assessments had undertaken specific training for every segment and regularly attended re-training sessions. They were constantly monitored and evaluated by experts for quality assurance [19].

### 2.3. Outcome Variables

Muscle strength, which was used as the indicator of frailty in this study, was assessed with the help of a grip strength test using a handgrip dynamometer (Takei digital handgrip dynamometer, Shinagawa-Ku, Tokyo, Japan). Participants performed the handgrip test in a standing position unless they had physical limitations, and each hand was tested three times. To increase precision and reliability, hand grip strength was assessed using maximum grip strength value in all trials. Handgrip strength was dichotomized, and the participants were categorized into “low grip strength” and “normal grip strength” groups. Three outcome variables were used: (i) Low grip strength indicated by grip strength <30 kg for men; <20 kg for women on the basis of earlier studies [20,21]; (ii) Low grip strenght indicated by grip strength <26 kg for men; <16 kg for women based on a previous study on a diverse sample of older adults [22]; and (iii) mean maximum grip strength which is the mean of the three-reading performed for the dominant hand.

This study used a higher cut off point for the outcome variable (grip strength <30 kg for men; <20 kg for women) as it is more appropriate for a relatively younger population. At the same time, lower cut off points (grip strength <26 kg for men; <16 kg for women) and a continuous variable (mean maximum grip strength) were also used as outcome variables for ensuring the sensitivity of the results.

### 2.4. Explanatory Variables

Periodontal disease was the exposure variable for the study. It was categorized into: (i) moderate/severe periodontitis; and (ii) mild/no periodontitis. Severe periodontitis was defined as being if the subject had 2 or more interproximal sites with Clinical Attachment Loss (CAL) > 6 mm, not necessarily present on the same tooth, or 1 or more interproximal sites with Probing Depth (PD) > 5 mm. Moderate periodontitis was defined as being if the subject had 2 or more interproximal sites with CAL > 4 mm, not necessarily present on the same tooth, or 2 or more interproximal sites with PD > 5 mm, not on the same tooth [23]. This definition of periodontitis was used as it is more appropriate in national surveys.

### 2.5. Covariates

Covariates consisted of demographic, socio-economic, lifestyle, behavioral and chronic medical conditions [10,12]. Demographic factors were age, gender, ethnicity/race (Hispanics “Mexican American and other Hispanics”, non-Hispanic Whites, non-Hispanic Blacks and other races). Socio-economic position was indicated using education and income levels. Income level was indicated by income-to-poverty ratio. Education level was recoded into four categories: (i) below high school (less than grade 9; and grades 9 to 11 including grade 12 without diploma); (ii) high school; (iii) college; and (iv) above college. Marital status was grouped into two categories: (i) singles (not married, divorced, widows/widowers, or separated); and (ii) couples (those living with a partner/spouse). Physical activity, which was self-reported, was categorized as “active” (at least 150 min for moderate-intensity exercise or equivalent other-intensity exercises) and “inactive” (less than the Physical Activity Guidelines for Americans) [24]. Smoking was grouped into: (i) current smoker (who has smoked >100 cigarettes in their lifetime and smokes at present); (ii) former smoker (smoked >100 cigarettes in their lifetime but has quit the habit now); and (iii) never smoker [19,25]. Systemic health included self-reported medical diagnosis of diabetes and other chronic medical conditions including hypertension, stroke, cardiovascular diseases, arthritis and cancer. Those individuals who had no systemic health condition were grouped as “no systemic diseases” and those who had one or more systemic health conditions were grouped as “having systemic diseases”. Diabetes was divided into two categories: (i) non-diabetic; and (ii) diabetic. BMI was used as a continuous variable in this study.

### 2.6. Data Analysis

Data analysis was done using STATA software. The overall distributions of all variables included in the analysis were assessed. The distribution of low grip strength within all co-variates was also assessed. Then logistic regression models were constructed to test the association between periodontal disease and two definitions of low grip strength. First, the unadjusted associations between moderate/severe periodontitis and grip strength were assessed. The second set of models were adjusted for age and gender, and finally the third set of models were adjusted for the rest of the covariates. For the third outcome variable (mean maximum grip strength), linear regression models were used. Three set of models were constructed similar to the first and second outcome variables.

## 3. Results

The major characteristics of the 1953 individuals who participated in the study and the percentage of older adults with a low grip strength are presented in Table 1. Out of the 1953 study participants, 47.2% were males. The prevalence of low grip strength (indicated by handgrip strength <30 kg for men, <20 kg for women) was 7.4% in men and 13.7% in women. While the average age of the study participants was 68.5 years, the low grip strength group had a mean age of 74.9 years. Among the study participants, 49.8% had moderate/severe periodontitis. The prevalence of low grip strength was 8.7% among people with no or mild periodontitis and 12.7% among those with moderate/severe periodontitis (Table 1).

The analyses of the association between periodontitis and low grip strength is presented in Table 2 (cut-off points: 30 kg for men, 20 kg for women). Periodontitis was significantly associated with low grip strength with odds ratio 1.53 (95% CI 1.03, 2.27, *p* < 0.05) in the unadjusted model. After adjusting for age and gender, this odds ratio was attenuated to 1.39 (95% CI 0.88, 2.19) and lost statistical significance (Table 2). In the fully adjusted model, the odds of having low grip strength was not significant at 1.09 (95% CI 0.67, 1.77). Of the other factors associated with frailty were factors such as age, income, marital status, BMI, physical activity, chronic medical conditions and diabetes (Table 2).

In terms of the association between periodontitis and the second variable of low grip strength (cut-off points: 26 kg for men, 16 kg for women), there were no significant associations in any of the models. In the fully adjusted model, those who had moderate or severe periodontitis had an odds ratio of 0.83 (95% CI 0.46, 1.5) of having low grip strength. Age and physical activity were the only factors which were significantly associated with this lower cut-off point of musculoskeletal frailty (Table 2).

Periodontitis was significantly associated with a lower mean of maximum grip strength in a model adjusted for age and gender, with a regression coefficient of −1.04 (95% CI −1.99, −0.09) (Table 3). The significance disappeared after adjusting for all the other variables in the fully adjusted model with a regression coefficient of −0.43 (95% CI −1.47, 0.60). Other variables which showed a significant association with maximum grip strength were age, gender, ethnicity, income, BMI, chronic medical conditions and diabetes (Table 3).

## 4. Discussion

The study assessed the association between moderate/severe periodontitis and handgrip strength among community-dwelling individuals aged ≥60 years in the USA. The main findings of the study showed that a definition of low grip strength (cut-off point: 30 kg for men, 20 kg for women) was significantly higher among people with moderate/severe periodontitis in the USA. It was found that the significance of the association disappeared after adjusting for age and gender. The findings suggest that the observed association could be attributed to older age and relevant common risk factors for periodontitis and frailty. Factors like age, income, marital status, BMI, physical activity and chronic medical conditions were significantly associated with low grip strength.

Periodontitis was also significantly associated with the mean of maximum grip strength after adjusting for age and gender. The significance was eliminated after accounting for socioeconomic and behavioral factors, and systemic conditions. This suggests that low grip strength among older adults can be directly influenced by age, income levels, and other socioeconomic conditions, which are linked to periodontitis. The findings of this study agree with a Mexican cross-sectional study, which suggested that age, income and social support could play a role in the relationship between frailty and oral health among elderly adults [26]. Although periodontitis was not statistically significant after adjusting for age and gender, it should be understood that the physiological process behind the initiation of periodontitis could be associated with frailty. Studies have shown that the energy imbalance which is related to severe periodontitis, has been associated with mobility loss and strength in the elderly [27], and high concentrations of pro-inflammatory cytokines are related to the change in inflammation state seen in frail patients [28]. Previous studies have shown that periodontal disease can affect the supporting structures of the teeth and could lead to tooth loss in elderly adults [16], and having worse dentition can significantly increase the risk of weakness and exhaustion in older adults [29]. A cross-sectional study conducted in Brazil showed that having fewer teeth can result in a higher chance of being frail [30]. Another study conducted among community-dwelling adults in the USA also showed that those with fewer teeth were at a significantly higher risk of being frail [12]. This link between periodontitis, tooth loss and frailty could possibly explain the reason why the prevalence of low handgrip strength was higher in adults with severe/moderate periodontitis when compared to those with no/mild periodontitis.

Previous studies that assessed the association between oral health and low hand grip strength focused on the number of teeth and the use of dental prothesis. A study that used NHANES found that older adults who have fewer than 20 teeth and do not use dentures have higher odds of musculoskeletal frailty indicated by low hand grip strength compared to older adults who have 20 teeth or more after adjusting for age and gender. They also found that poor nutritional intake mediated this association [12]. Similar to the results of this study, the significant association between oral health and low hand grip strength lost significance in the fully adjusted model. Yun and colleagues found that Korean older adults who have fewer than nine teeth, and those who use complete dentures have higher odds of having low handgrip strength compared to older adults who have more than 20 teeth, and those who use fixed prothesis, respectively. It is important to note that these associations were significant for older men, but were not significant for older women [13]. Furthermore, hand grip strength was associated with several oral function indicators among Japanese older adults [14]. To the best of our knowledge, this is the first study that has assessed the association between low hand grip strength and periodontal disease among older adults. The results of this study are in line with these studies and indicate that oral health and, to a lesser degree, periodontal disease, is associated with low handgrip strength among older adults. This highlights the importance of maintaining oral health at old age and the importance of incorporating oral health measures and interventions among multidisciplinary interventions for improving and maintaining physical status and frailty for the ageing population.

Marital status was also found to have a significant association with low grip strength. The prevalence of low grip strength was lower among couples when compared to those who were single. A cohort study [31] had previously suggested that males could benefit from marriage because of its protective effects against low grip strength. However, in the current study, females had a higher prevalence of low grip strength than males. A similar pattern was seen in another study which used data from a Cardiovascular Health study where females had a higher prevalence of frailty than males [11]. It is possible that women are older than men in these studies. Physical activity was another factor which was significantly associated with low grip strength. Inactive older adults were at greater risk of having low grip strength than active older adults. Previous research has established a strong link between physical activity and frailty. Furthermore, along with hand grip strength, physical activity has also been used as a frailty marker [11]. Previous research has also shown that weak grip strength and physical inactivity are common characteristics seen in frail older adults [32]. There is also evidence that participation in physical activities can delay the onset as well as the progression of frailty [33].

It was also observed in this study that the prevalence of low grip strength was substantially higher among older adults with chronic medical conditions including diabetes. Comorbid conditions and diabetes were significantly associated with low grip strength. Earlier research has shown that chronic medical conditions, which usually coexist as comorbidities, contributes to the development of frailty [34]. Our study also suggests that comorbidity is strongly related to the onset of frailty and the association between periodontal disease and low grip strength could potentially be attributed to comorbid conditions.

To the best of our knowledge, this is the first study to examine the relationship between periodontal disease and low grip strength using a nationally representative sample of older adults in the USA. Recruiting study participants from a nationally representative community-dwelling older population is a major strength of this study as comparisons could be made with the general older adult population. We have also used different cut-off points for low grip strength based on previous systematic reviews [20,21] as well as studies conducted on a diverse cohort of older adults [22]. The original continuous variable of low grip strength—maximum grip strength—was also used as an outcome variable in the analysis. One of the major limitations of this study is that even though samples were representative of the general population, it is difficult to make causal inferences due to the cross-sectional nature of the study. Excluding institutionalized older adults from the NHANES study could have resulted in the omission of many older adults who were potentially frail and have a low grip strength.

## 5. Conclusions

This study examined the association between moderate/severe periodontal disease and grip strength on a nationally representative sample of community-dwelling older adults in the USA using two NHANES surveys (2011–2012 and 2013–2014). The study showed that low grip strength was higher among people with moderate/severe periodontitis. To enhance our understanding of the association between periodontitis and grip strength, there is a need to perform longitudinal studies. While the significant relationship between periodontitis and maximum grip strength persisted after adjusting for age and gender, it disappeared when adjusted for the remaining variables. As a result, it should be concluded that the observed association could be due to the common risk factors for periodontitis and low grip strength, especially comorbidity. Our study emphasizes the need of further research in this area to examine the association of periodontitis, comorbid conditions and the onset of frailty.

## Figures and Tables

**Table 1:** Distribution of variables and percentage of low grip strength “cut-off points: 26 kg for men, 16 kg for women” prevalence among the 1953 survey participants aged 60 and older, National Health and Nutrition Examination Survey 2011–2014, USA.

Variable	Total Sample Percentage/Mean (95% CI)	Percentage/Mean of Participants with Low Grip Strength (95% CI)	Significance (*p*-Value *)
**Gender (%)**			
Male	47.2 (44.3, 50.1)	7.4 (5.7, 9.5)	0.000
Female	52.7 (49.8, 55.7)	13.7 (11.2, 16.4)	
**Age in years (mean)**	68.5 (67.9, 69.0)	74.9 (74.0, 75.8)	0.000
**Ethnicity (%)**			
Hispanic	7.3 (4.8, 11.1)	15.5 (10.7, 21.9)	0.039
Non-Hispanic White	79.4 (75.4, 82.9)	10.2 (8.3, 12.4)	
Non-Hispanic Black	7.8 (5.8, 10.5)	9.9 (6.7, 14.4)	
Other Race	5.3 (3.7,7.5)	12.5 (7.8, 19.4)	
**Ratio of family income to poverty (mean)**	3.1 (2.9, 3.2)	2.2 (1.9, 2.4)	0.000
**Education level (%)**			
Grade school or Less	13.1 (10.4, 16.3)	17.5 (13.2, 22.9)	0.001
High school	21.0 (18.1, 24.3)	12.2 (9.0, 16.3)	
College	32.6 (29.4, 35.9)	10.1 (7.8, 12.8)	
Above College	33.1 (28.8, 37.7)	7.7 (5.7, 10.2)	
**Marital status (%)**			
Living with partner/married	66.9 (64.5, 69.3)	7.2 (5.5, 9.3)	
Single	33.0 (30.6, 35.4)	17.8 (14.7, 21.5)	0.000
**Smoking status (%)**			
Never smoker	53.0 (49.9, 56.1)	12.5 (10.1, 15.5)	0.164
Former smoker	37.1 (34.1, 40.1)	8.5 (6.6, 11.0)	
Current smoker	9.8 (8.1, 11.7)	8.8 (3.9, 18.8)	
**Diabetes (%)**			
Absent	71.2 (67.6, 74.5)	9.4 (7.7, 11.6)	
Present	28.7 (25.4, 32.4)	13.8 (11.8, 16.0)	0.001
**Comorbid condition (%)**			
No chronic disease	35.9 (32.2, 39.7)	6.3 (4.7, 8.3)	
Having chronic diseases	64.0 (60.2, 67.7)	13.2 (11.0, 15.6)	0.000
**Body Mass Index (kg/m^2^) (mean)**	28.7 (28.3, 29.2)	26.9 (25.8, 28.1)	0.004
**Physical activity (%)**			
Active	65.7 (62.1, 69.0)	7.6 (5.9, 9.9)	
Inactive	34.3 (30.9, 37.8)	16.5 (13.1, 20.6)	0.000
**Periodontitis**			
No or mild	50.1 (45.7, 54.6)	8.7 (6.3,11.8)	
Moderate/severe	49.8 (45.3,54.3)	12.7 (11.0, 14.7)	0.034

Notes: CI = confidence interval, * *p*-value based on chi-square for categorical variables and *t*-test for numerical variables.

**Table 2:** Logistic regression models showing the associations between Periodontal disease and low grip strength among the study sample (N = 1953).

		Low Grip Strength Cut-Off Points: 30 kg for Men, 20 kg for Women			Low Grip Strength Cut-Off Points: 26 kg for Men, 16 kg for Women		
		Model 1	Model 2	Model 3	Model1	Model 2	Model3
		OR (95% CI)	OR (95% CI)	OR (95% CI)	OR (95% CI)	OR (95% CI)	OR (95% CI)
**Periodontitis**	No or mild	(Reference)					
	Moderate/Severe	1.53 * (1.03, 2.27)	1.39 (0.88, 2.19)	1.09 (0.67, 1.77)	1.24 (0.67, 2.27)	1.03 (0.58, 1.85)	0.83 (0.46, 1.50)
**Age**			1.19 *** (1.15, 1.22)	1.17 *** (1.13, 1.20)		1.19 *** (1.12, 1.25)	1.15 *** (1.07, 1.24)
**Gender**	Male	(Reference)					
	Female		2.10 ** (1.34, 3.30)	1.41 (0.81, 2.46)		1.57 (0.83, 2.97)	1.01 (0.40, 2.53)
**Ethnicity**	Non-Hispanic White	(Reference)					
	Hispanic			1.31 (0.63, 2.74)			0.77 (0.26, 2.20)
	Other race			0.87 (0.40, 1.86)			0.86 (0.28, 2.66)
	Non-Hispanic black			0.51 (0.26, 1.03)			0.57 (0.20, 1.65)
**Marital status**	Single	(Reference)					
	Living with partner			0.56 * (0.34, 0.92)			0.51 (0.21, 1.22)
**Income**	(ratio of family income to poverty)			0.78 ** (0.69, 0.89)			0.82 (0.64, 1.04)
**Education**	Above college	(Reference)					
	College			1.10 (0.68, 1.76)			1.20 (0.49, 2.93)
	High school			1.05 (0.65, 1.69)			1.30 (0.49, 3.44)
**Smoking status**	Never smoker	(Reference)					
	Former smoker			0.72 (0.48, 1.07)			0.69 (0.33, 1.42)
	Current smoker			1.29 (0.42, 3.87)			1.04 (0.21, 5.16)
**Diabetes**	No	(Reference)					
	Yes			1.62 * (1.13, 2.34)			2.09 (0.96, 4.51)
**Comorbidity**	No systemic diseases	(Reference)					
	Having systemic disease			1.53 * (1.07, 2.19)			1.30 (0.84, 2.01)
**BMI (kg/m^2^)**	Mean BMI			0.95 * (0.92, 0.99)			0.95 (0.90, 1.00)
**Physical activity**	Inactive	(Reference)					
	Active			0.59 * (0.39, 0.89)			0.45 * (0.22, 0.89)

Notes: CI = confidence interval; OR = odds ratio. The *p* values for the OR were obtained using logistic regression: * *p* < 0.05; ** *p* < 0.01; *** *p* < 0.001. Model 1: Unadjusted Logistic regression model. Model 2: Adjusted for age and gender. Model 3: Fully adjusted (as in Model 2 and additionally adjusted for ethnicity, income, education level, marital status, smoking status, diabetes, comorbidity, BMI and physical activity).

**Table 3:** Linear regression models showing the associations between Periodontal disease and low grip strength among the study sample (*N* = 1953).

		Maximum Grip Strength as Outcome Variable		
		Model 1	Model 2	Model 3
		Coefficient (95% CI)	Coefficient (95% CI)	Coefficient (95% CI)
**Periodontitis**	No or mild	(Reference)		
	Moderate/Severe	0.61 (−0.87, 2.10)	−1.04 * (−1.99, −0.09)	−0.43 (−1.47, 0.60)
**Age**			−0.44 *** (−0.49, −0.39)	−0.83 *** (−0.43, −0.33)
**Gender**	Male	(Reference)		
	Female		−15.60 *** (−16.53, −14.67)	−15.02 *** (−16.14, −13.91)
**Ethnicity**	Non-Hispanic White	(Reference)		
	Hispanic			−0.14 (−1.42, 1.14)
	Other race			0.86 (−1.07, 2.80)
	Non-Hispanic black			4.36 *** (3.02, 5.70)
**Marital status**	Single	(Reference)		
	Living with partner			0.66 (−0.34, 1.66)
**Income**	(ratio of family income to poverty)			0.49 *** (0.25, 0.73)
**Education**	Above college	(Reference)		
	College			0.65 (−0.64, 1.94)
	High school			−0.11 (−1.04, 0.81)
**Smoking status**	Never smoker	(Reference)		
	Former smoker			0.45 (−0.40, 1.31)
	Current smoker			−0.13 (−1.30, 1.02)
**Diabetes**	No	(Reference)		
	Yes			−1.21 ** (−1.99, −0.44)
**Comorbidity**	No systemic diseases	(Reference)		
	Having systemic disease			−0.94 * (−1.71, −0.17)
**BMI (kg/m^2^)**	Mean BMI			0.13 *** (0.08, 0.19)
**Physical activity**	Inactive	(Reference)		
	Active			1.05 (0.26, 1.84)

Notes: CI = confidence interval. The *p* values for the coefficients were obtained using linear regression: * *p* < 0.05; ** *p* < 0.01; *** *p* < 0.00. Model 1: Unadjusted Logistic regression model. Model 2: Adjusted for age and gender. Model 3: Fully adjusted (as in Model 2 and additionally adjusted for ethnicity, income, education level, marital status, smoking status, diabetes, comorbidity, BMI and physical activity).
